# *In vivo *natural killer and natural killer T-cell depletion affects mortality in a murine pneumococcal pneumonia sepsis model

**DOI:** 10.1186/cc10614

**Published:** 2012-03-20

**Authors:** E Christaki, E Diza, SM Opal, A Pistiki, DI Droggiti, DP Carrer, M Georgitsi, N Malisiovas, P Nikolaidis, EJ Giamarellos-Bourboulis

**Affiliations:** 1 Aristotle University of Thessaloniki, Greece; 2 Memorial Hospital of RI, Alpert School of Medicine of Brown University, Providence, RI, USA; 3 University of Athens, Medical School, Athens, Greece

## Introduction

Apart from macrophages and neutrophils, natural killer (NK) and natural killer T (NKT) cells have been found to play a role in the early stages of bacterial infection. In this study, we investigated the role of NK and NKT cells in host defense against *Streptococcus pneumoniae*, using a murine pneumococcal pneumonia sepsis model. Our hypothesis was that NK and NKT cells play an immune-regulatory role during sepsis and thus *in vivo *depletion of those cell populations may affect mortality.

## Methods

We used four groups of C57BL/6 mice (A, B, C and D, *n *= 10 mice/group). Animals were infected intratracheally with 50 μl of *S. pneumoniae *suspension (10^6 ^cfu). Twenty-four hours prior to bacterial inoculation, Group A received 50 μl of anti-asialoGM1 rabbit polyclonal antibody (Wako Chemicals GmbH, Neuss, Germany) intravenously (i.v.) to achieve *in vivo *NK cell inactivation; in Group B, NKT cell depletion was performed by targeting the CD1d receptor using 2 mg/kg of the monoclonal antibody anti-CD1d, clone 1B1 (BD Pharmingen, San Diego, CA, USA) i.v.; Group C (control) received an equivalent amount of isotype antibody control (nonspecific Ig). Group D received sham intratracheal installation of normal saline. Animals were observed daily for 7 days and deaths were recorded. The survival analysis was plotted using the Kaplan-Meier method and differences in survival between groups were compared with the log-rank test.

## Results

**Figure 1:**
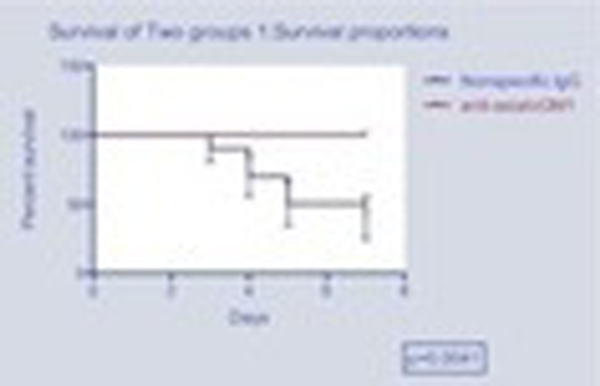
**Kaplan-Meier survival curve of groups A and C**.

**Figure 2:**
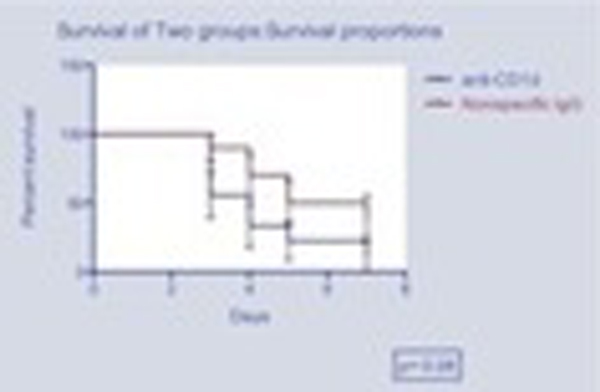
**Kaplan-Meier survival curve of groups B and C**.

We found that *in vivo *NK cell depletion improved survival after pneumococcal pneumonia and sepsis in the group of mice that received the anti-asialoGM1 antibody when compared with animals that received nonspecific IgG antibody (*P *= 0.041) (Figure 1). Nevertheless, when NKT cell depletion was attempted, survival worsened compared to the control group; however, that difference did not reach statistical significance (*P *= 0.08) (Figure 2).

## Conclusion

Our study has shown that NK cells appear to contribute to mortality in pneumococcal pneumonia. More research is needed to explore their role in host response to bacterial infection and sepsis.

